# Use of Fall-Risk Inducing Drugs in Patients Using Anti-Parkinson Drugs (APD): A Swedish Register-Based Study

**DOI:** 10.1371/journal.pone.0161246

**Published:** 2016-08-18

**Authors:** Ylva Haasum, Johan Fastbom, Kristina Johnell

**Affiliations:** Aging Research Center, Department of Neurobiology, Care Sciences and Society, Karolinska Institutet and Stockholm University, Stockholm, Sweden; Oslo Universitetssykehus, NORWAY

## Abstract

**Objectives:**

Many drugs increase the risk of falls in old age. Although persons with Parkinson’s disease (PD) are at increased risk of experiencing falls and fractures, the use of fall-risk inducing drugs (FRIDs) in this population has not previously been investigated. The objective of this study was to investigate the burden of use of FRIDs in older persons treated with anti-Parkinson drugs (APD; used as a proxy for PD), compared to persons without APD.

**Methods:**

We analyzed individual data on age, sex, type of housing and drug use in 1 346 709 persons aged ≥ 65 years in *the Swedish Prescribed Drug Register* on the date of 30 September 2008. Main outcome measure was the use of FRIDs.

**Results:**

FRIDs were used by 79% of persons with APD and 75% of persons without APD. Persons with APD were more likely to use ≥ 1 FRIDs compared to persons without APD (adjusted OR: 1.09; 95% CI: 1.06-1-12). The association was stronger for concomitant use of ≥ 5 FRIDS (adjusted OR: 1.49; 95% CI: 1.44–1.55).

**Conclusions:**

The high use of FRIDs among persons with APD indicates that these patients may be at increased risk of drug-induced falls. Further studies are needed to investigate how these drugs affect the risk of falling in persons with PD.

## Introduction

Falls occur frequently in the elderly population and injuries related to falls is a common cause of emergency department visits, hospitalization and premature admittance to nursing homes [1–4]. It has been estimated that approximately 30% of community-dwelling and more than 50% of institutionalized older persons fall at least once a year and around half of the persons who fall are recurrent fallers [5, 6]. In Sweden, approximately 40 000 falls among people aged ≥ 65 years led to hospitalization in 2010, an increase by around 8% since 2001 [1]. People with Parkinson’s disease (PD), the second most common neurodegenerative disorder after Alzheimer’s disease, have an increased risk of falling, both compared to healthy controls and compared to persons with other neurological diseases [7–9]. Nearly 70% of PD patients fall annually and 50% of them fall more than once annually [10]. In a worldwide study, falls and fractures were one of the most common causes for hospitalization of PD patients [11]. Falls may lead to severe injuries such as hip-fracture, but also less severe falls may lead to a restriction in daily activities due to fear of experiencing another fall [8]. Thus, falls may have significant impact for the individual and also impose substantial economic burden for the society [4].

One modifiable risk factor for falls among elderly people is the use of drugs. Polypharmacy, often defined as concomitant use of ≥5 drugs, is common in old age and has been associated with increased risk of falls, hospital admissions and fractures [12–14]. However, not only the number of drugs but also the type of drugs influence the risk of falling [12, 14]. Numerous fall-risk inducing drugs (FRIDs) have been identified, including several cardiovascular drugs, psychotropics (e.g. benzodiazepines, antidepressants and antipsychotics), opioids and anticholinergics [12, 15–19].

Falls in PD patients has been extensively studied and several factors related to the disease, such as freezing in gait, postural instability, muscle weakness, impaired balance and impaired cognition, have been suggested [20, 21]. However, to our knowledge no study has so far investigated how widespread the use of FRIDs is in this population with an already increased risk of falls. Therefore, this study aims to investigate the use of FRIDs, in older persons treated with anti-Parkinson drugs (APD; used as a proxy for PD), compared to persons not using APD.

## Methods

### Study population

We analyzed data on age, sex, and drug use in 1 346 709 elderly persons aged ≥ 65 years registered in the *Swedish Prescribed Drug Register* (SPDR) in July to September 2008. The SPDR contains information about all prescription drugs dispensed at Swedish pharmacies to the entire Swedish population (about 9 million inhabitants) [22]. Via record linkage to the *Swedish Social Services Register*, we also retrieved information about type of housing (i.e. home dwelling / institution). Since 2007, all Swedish municipalities report individual-based information of institutional care to this register [23]. Almost all institutional care in Sweden is organized and granted by the municipalities.

We calculated a 1-day point prevalence for drug use on the arbitrarily chosen date of September 30, 2008. The method for the calculations has been described in detail elsewhere [24]. Briefly, since prescription drugs are dispensed for at most 3 months in Sweden, we used information about when the prescription was filled, the amount of drugs dispensed and prescribed dosage, from the preceding 3 months to calculate the 1-day point prevalence. If the same drug was dispensed more than once during the 3 month period, it was calculated as one drug.

### Definitions

The Anatomical Therapeutic Chemical (ATC) classification system was used for classification of drugs, as recommended by the Word Health Organization [25].

APD usage, defined as use of any dopaminergic anti-Parkinson drug in ATC-class N04B (i.e. Dopa and dopa derivatives (ATC-code N04BA), adamantane derivatives (N04BB), dopamine agonists (N04BC), monoamine oxidase B inhibitors (N04BD) and other dopaminergic agents (N04BX)) was used as a proxy for PD [26].

The following drug classes that may increase the risk of falls in old people (i.e. FRIDs) were analyzed: opioids (ATC-code N02A), antipsychotics (N05A (N05AN excluded)), anxiolytics (N05B), hypnotics and sedatives (N05C), antidepressants (N06A), vasodilators used in cardiac diseases (C01D), antihypertensives (C02), diuretics (C03), beta-blocking agents (C07), calcium channel blockers (C08), agents acting on the renin-angiotensin system (C09), alpha-adrenoreceptor antagonists (G04CA), dopaminergic anti-parkinson drugs (N04B) [27] and anticholinergic drugs [19]. We used a list of anticholinergic drugs published by the Swedish National Board of Health and Welfare: anticholinergic drugs for functional gastrointestinal disorders (A03AB, A03BA, A03BB), anticholinergic antiemetics (A04AD), antiarrhythmic s (C01BA), drugs for urinary frequency and incontinence (G04BD), opioids in combination with antispasmodics (N02AG), anticholinergic anti-parkinson drugs (N04A), some antipsychotics (N05AA, N05AF03, N05AH02), hydroxyzine (N05BB01), non-selective monoamine reuptake inhibitors (N06AA) and certain antihistamines (R06AA02, R06AB, R06AD, R06AX02) [27]. Hence, the use of any FRIDs in this study refer to the use of at least one drug in the above mentioned drug classes with the exception of anti-parkinson drugs in ATC class N04A and N04B, since we chose to analyze these drugs separately. The concomitant use of several FRIDs refer to the total number of used drugs in the ATC classes defined above, i.e. it can be drugs from different ATC classes or different drugs from the same ATC class. The use of specific FRIDs refer to the use of at least one drug in the respective ATC classes described above. The use of any anticholinergics refers to use of any of the anticholinergics listed above (ATC class N04A excluded).

We also investigated the use of osteoporosis drugs (use of drugs in ATC classes A12AX, G03XC01, M05BA or M05BB), and polypharmacy, defined as use of ≥ 5 drugs simultaneously.

The type of housing variable was defined as home-dwelling (living in own home) or institutionalized (e.g. nursing-home, sheltered accommodation) [24].

### Statistical analysis

We performed both crude and multivariate logistic regression analysis in order to study whether use of APD was associated with use of FRIDS. In model 1, adjustment was made for age (continuous variable) and sex. In model 2, additional adjustment was made for type of housing.

The results are presented as odds ratios with 95% confidence intervals. SPSS 22.0 for Windows (SPSS Inc., Chicago, IL) was used for the analyses.

### Ethical considerations

The study was approved by the regional ethical review board in Stockholm (Dnr 2009/477-31/3). Only de-identified register-based data were used.

## Results

In this study of 1 346 709 elderly persons, 24 633 persons used APD, which corresponds to 1.8% of the study population. Persons with APD were on average older (78 compared to 76 years), used more drugs (on average 7.2 drugs compared to 4.5 drugs per person), and more often lived in institutions (16% compared to 6%) than persons not using APD (Table 1). Persons with APD used on average 1.4 APD per person (SD: 0.77).

**Table 1:** Basic characteristics of the study population according to APD^a^ user status.

	APD^a^ non-user n = 1322076	APD^a^ user n = 24633
Age, mean (SD)	76.2 (7.9)	77.7 (7.4)
Women, n (%)	760816 (57.5)	13743 (55.8)
Institution, n (%)	82345 (6.2)	3967 (16.1)
Number of drugs, n mean, (SD)	4.5 (3.4)	7.2 (4.0)

^a^ APD = anti-parkinson drugs

In total, 79% of persons with APD and 75% of persons without APD were exposed to at least one FRID. Table 2 shows use of FRIDs according to APD user status. Both the mean number of FRIDs (2.2 FRIDs per person in persons with APD compared to 1.8 in persons without APD) and the proportion who used several FRIDs (e.g. 12% used 5 or more FRIDS among persons with ADP compared to 7% among persons without APD) was higher among persons with APD. Also the pattern of use of specific FRIDs differed in persons with and without APD, with a higher proportion of persons with APD using psychotropic drugs and anticholinergic drugs. Most of the cardiovascular drugs were used less frequently by persons with APD. A separate analysis of the use of anticholinergic APDs showed that 0.6% of the persons with APD and 0.2% without APD used this drugs.

**Table 2:** Use of Fall-risk inducing drugs according to APD^a^ user status.

Characteristic	ATC class	APD^a^ non-user (n = 1322076)	APD^a^ user (n = 24633)
Fall-risk inducing drugs, count, n mean (SD)		1.83 (1.68)	2.19 (1.92)
Number of used fall-risk inducing drugs, n (%)			
0		329410 (24.9)	5244 (21.3)
1		329421 (24.9)	5484 (22.3)
2		279005 (21.1)	4718 (19.2)
3		184629 (14.0)	3650 (14.8)
4		102488 (7.8)	2551 (10.4)
5 or more		97123 (7.3)	2986 (12.1)
Specific fall-risk inducing drugs, n (%)			
Opioids	N02A	97244 (7.4)	3748 (15.2)
Antipsychotics	N05A^b^	32473 (2.5)	1418 (5.8)
Anxiolytics	N05B	94382 (7.1)	3364 (13.7)
Hypnotics and sedatives	N05C	207034 (15.7)	6286 (25.5)
Antidepressants	N06A	157507 (11.9)	6345 (25.8)
Vasodilators used in cardiac diseases	C01D	97560 (7.4)	2143 (8.7)
Antihypertensives	C02	8480 (0.6)	128 (0.5)
Diuretics	C03	372521 (28.2)	7318 (29.7)
Beta blocking agents	C07	438557 (33.2)	6291 (25.5)
Calcium channel blockers	C08	233582 (17.7)	2997 (12.2)
Agents acting on the renin-angiotensin system	C09	401700 (30.4)	5705 (23.2)
Alpha-adrenoreceptor antagonists	G04CA	29898 (2.3)	698 (2.8)
Anticholinergic drugs^c^	see methods	69267 (5.2)	2809 (11.4)

^a^ APD = anti-parkinson drugs

^b^ N05AN is excluded from N05A

^c^ N04A is excluded from anticholinergic drugs

Results from the logistic regression analyses are shown in Table 3. We found a modestly increased probability of being exposed to any FRIDs in persons with APD compared to persons without APD (OR: 1.09; 95% CI: 1.06-1-12) after adjustment for age, sex and type of housing. The association between use of APD and use of ≥5 FRIDs was stronger (OR: 1.49; 95% CI: 1.44–1.55), after adjustment for age, sex and type of housing. Of the specific FRIDs, persons with APD were more likely to be exposed to all types of psychotropics, with the strongest association found for use of antidepressants (OR: 2.19; 95% CI: 2.13–2.26) after adjustment for age, sex and type of housing. Use of APD was also positively associated with use of opioids, vasodilators used in cardiac diseases, alpha-adrenoceptor antagonists and anticholinergics. However, after adjustment for age, sex and type of housing, persons with APD were less likely to use most cardiovascular FRIDs, i.e. beta-blocking agents, calcium channel blockers, agents acting on the renin-angiotensin system, antihypertensives and diuretics (borderline significance for the two latter drug classes).

**Table 3:** Odds ratios with 95% confidence intervals for use of fall-risk inducing drugs in persons using APDs^a^ compared to persons not using APD^a^.

	ATC class	Crude	Model 1^b^	Model 2^c^
Use of any fall-risk inducing drug		1.23 (1.19–1.27)	1.14 (1.10–1.17)	1.09 (1.06–1.12)
Use of 5 or more fall-risk inducing drug		1.74 (1.67–1.81)	1.64 (1.58–1.71)	1.49 (1.44–1.55)
Use of specific fall-risk inducing drug				
Opioids	N02A	2.26 (2.18–2.34)	2.19 (2.11–2.27)	2.01 (1.94–2.09)
Antipsychotics	N05A^d^	2.43 (2.30–2.56)	2.30 (2.18–2.43)	1.46 (1.38–1.55)
Anxiolytics	N05B	2.06 (1.98–2.13)	1.99 (1.92–2.07)	1.71 (1.65–1.78)
Hypnotics and sedatives	N05C	1.85 (1.79–1.90)	1.78 (1.73–1.84)	1.68 (1.63–1.73)
Antidepressants	N06A	2.57 (2.49–2.64)	2.53 (2.45–2.60)	2.19 (2.13–2.26)
Vasodilators used in cardiac diseases	C01D	1.20 (1.14–1.25)	1.10 (1.05–1.15)	1.11 (1.07–1.17)
Antihypertensives	C02	0.81 (0.68–0.96)	0.80 (0.67–0.95)	0.84 (0.70–1.00)
Diuretics	C03	1.08 (1.05–1.11)	1.00 (0.97–1.03)	0.97 (0.95–1.00)
Beta blocking agents	C07	0.69 (0.67–0.71)	0.67 (0.65–0.69)	0.70 (0.68–0.72)
Calcium channel blockers	C08	0.65 (0.62–0.67)	0.64 (0.61–0.66)	0.67 (0.64–0.69)
Agents acting on the renin-angiotensin system	C09	0.69 (0.67–0.71)	0.69 (0.67–0.71)	0.72 (0.70–0.74)
Alpha-adrenoreceptor antagonists	G04CA	1.26 (1.17–1.36)	1.17 (1.09–1.27)	1.20 (1.11–1.30)
Anticholinergic drugs^e^	see methods	2.33 (2.24–2.42)	2.32 (2.22–2.41)	2.13 (2.04–2.21)

^a^ APDs = anti-Parkinson drugs

^b^ Adjusted for age (continuous) and sex

^c^ Additional adjustment for type of housing (own home / institution)

^d^ N05AN is excluded from N05A

^e^ N04A is excluded from anticholinergic drugs

We also analyzed polypharmacy (i.e. use of ≥5 drugs) and found that 71% of persons with APD and 42% of persons without APD were exposed to polypharmacy. Furthermore, 12.3% of persons with and 8.9% of persons without APD used osteoporosis drugs. After adjustment for age, sex and type of housing, persons with APD were more likely to be exposed to polypharmacy (OR: 3.13; 95% CI: 3.05–3.22), and to use osteoporosis drugs (OR: 1.44; 95% CI: 1.39–1.50) compared with persons without APD.

## Discussion

### Main findings

The prevalence of use of FRIDs other than APDs was high in this study (used by 79% and 75% in persons with and without APD, respectively). A high use of FRIDs was also found in a previous Swedish study of older persons who used multi-dose drug dispensing, where nearly 90% were exposed to at least one FRID [28]. In another Swedish study, about 58% of the community dwelling elderly persons who experienced an injurious fall used FRIDs compared to about 36% among persons who did not experience an injurious fall [14]. Moreover, we found that persons with APD were more likely to be exposed to several FRIDs concurrently. Concomitant use of several FRIDs may put elderly people at high risk of experiencing injurious falls as the risk increases with increasing number of FRIDs [3, 14]. Bennet et al. found that the fall-risk increased with 70% for each additionally used FRID [3].

Of the specific FRIDs, we found an association between APD and use of all analyzed psychotropic drug classes, opioids, vasodilators used in cardiac diseases and alpha-adrenoreceptor antagonists, which remained significant after adjustment for age, sex and type of housing. The higher use of some of these drugs in persons with APD may be explained by the high prevalence of psychiatric disorders and pain in this population. It has been estimated that 20–40% of PD patients suffer from major depression [29], 25–43% from various anxiety disorders [30], and sleep-disturbances is also very common [31]. PD patients may also experience hallucinations, especially in the later stages of the disease, which is a strong predictor for admittance to nursing home in this population [32]. Various types of pain also occur frequently in PD patients [33]. However, use of central nervous system drugs, especially psychotropics, increases the risk of falls [12], and PD patients with an already higher risk of falling may be particularly susceptible. Also, the evidence for the effectiveness of use of many of the psychotropic drugs is limited in the treatment of non-motor symptoms in PD [34]. Psychiatric drugs may not even be as effective in PD patients as in the general population [35]. It is important that these drugs are not prescribed in a routine like manner and that regular reevaluations of the drug treatment are being performed.

Several of the analyzed FRIDs may cause or worsen orthostatic hypotension in elderly people, particularly the cardiovascular FRIDs, dopaminergic anti-parkinson drugs, antipsychotics, antidepressants and alpha-adrenoreceptor antagonists [27]. Orthostatic hypotension can lead to fainting and falls in older people [27]. PD patients often experience orthostatic hypotension caused by PD dysautonomia and/or drugs [36]. In this study, persons with APD used on average 1.4 APD per person. Although APD can reduce the fall-risk in some PD patients, since levodopa can improve symptoms of freezing of gait and gait variability [37, 38], dopaminergic drugs and levodopa is also an often overlooked risk factor for orthostatic hypotension [36, 39]. Analysis of the specific cardiovascular FRIDs revealed that persons with APD were less likely to use most types of cardiovascular drugs, after adjustment for age, sex and type of housing. However, the percentage using these drugs was high in both persons with and without APD. Also, diuretics were the most commonly used FRID among persons with APD, and use of diuretics has been strongly associated with orthostatic hypotension in PD patients [36].

Our analysis of anticholinergic drugs showed that these drugs were more than twice as common among persons with APD as without APD (i.e. used by 11% and 5% respectively, anticholinergic anti-parkinson drugs excluded). Anticholinergic drugs are often used by persons with PD for treatment of non-motor symptoms such as mood disorders, urinary bladder dysfunction and pain. However, use of anticholinergics may increase the risk of injurious falls in elderly people. [19]. Anticholinergics also have cognitive side effects. This may be particularly detrimental to persons with PD because many of them suffer from cognitive impairment. Ehrt et al. found an association between use of anticholinergics and cognitive decline in persons with PD [40]. Also, a recent study of persons with PD found that those with the highest anticholinergic burden were at increased risk of experiencing adverse health outcomes such as fractures, delirium and admission to emergency departments [41].

We also found a strong association between use of APD and polypharmacy. Polypharmacy has been associated with orthostatic hypotension and falls in the elderly population [36, 42, 43]. Also, persons with APD were more likely to use osteoporosis drugs. This may indicate a higher fracture rate in persons with APD compared to persons without APD since use of osteoporosis drugs and overall drug use may increase after a hip fracture [44, 45].

### Limitations

The cross-sectional design of this study does not allow us to draw conclusions about causality. The SPDR does not contain information about comorbidities or underlying diagnoses. In this study we have assumed that patients treated with APD have PD [26]. However, some patients may be treated with APD for other diagnoses, e.g. other types of Parkinsonism, restless legs and multiple system atrophy [26, 46, 47]. Thereby, APD usage may overestimate the prevalence of PD, especially among women because of increased use of APD for restless legs [47]. The prevalence of PD may also be underestimated because there may be a time-gap from onset of the disease until APD treatment is initiated [48]. Hence, PD patients not yet receiving treatment with APD will not be included in this study. Also, we did not analyze the anticholinergic anti-Parkinson drugs (ATC class N04A), since these drugs are often used for other indications than PD in Sweden. Only rarely are PD patients treated with anticholinergic PD drugs alone for a long time, particularly among older people.

Although we were not able to adjust our analysis for comorbidity, we took into account the type of housing. As frailty and morbidity is very common in institutions [49], institutionalization may be a rough measure of frailty and morbidity in the elderly population.

We analyzed drug use in 1 346 709 persons aged 65 years and older registered in the SPDR during July to September 2008. This corresponded to about 81% of the total population in this age group (according to Statistics Sweden’s census data from September 30, 2008). Only information about dispensed drugs are recorded in the SPDR. Data on over-the-counter drugs and drugs supplied from drug store rooms in institutions are not included. This may have led to an underestimation of drug use.

Since prescribed drugs are dispensed for a maximum of 90 days’ supply in Sweden, we assumed that all currently used drugs would be dispensed during the prior 3 months. However, if a drug was dispensed before the 3-month period but used at a slower rate than intended, it was not included in this study. We might also have counted drugs dispensed during the 3-month period but discontinued prematurely [24].

In conclusion, a high percentage of older people in Sweden are exposed to FRIDs. Persons with APD were at higher risk of being exposed to FRIDs, in particular to concomitant use of several FRIDs. This was mainly explained by the high use of psychotropics in this population. Given that use of FRIDs, and in particular several FRIDs concurrently, increases the risk of injurious falls in old age, our results indicate that persons with APD may be at increased risk of drug-induced falls. Efforts to reduce the use of FRIDs may be an important step to decrease the risk of falls among PD patients. However, further studied are needed focusing on fall-risk in relation to use of FRIDs in persons with PD.
